# Complementary and alternative medicine use in adults with autism spectrum disorder in Germany: results from a multi-center survey

**DOI:** 10.1186/s12888-019-2043-5

**Published:** 2019-02-01

**Authors:** Juliana Höfer, Falk Hoffmann, Inge Kamp-Becker, Charlotte Küpper, Luise Poustka, Stefan Roepke, Veit Roessner, Sanna Stroth, Nicole Wolff, Christian J. Bachmann

**Affiliations:** 1 Department of Health Services Research, Carl von Ossietzky University Oldenburg, Ammerländer Heerstraße 140, 26129 Oldenburg, Germany; 2 Department of Child and Adolescent Psychiatry, Psychosomatics and Psychotherapy, Philipps University Marburg, Hans-Sachs-Str. 4, 35039 Marburg, Germany; 3 Department of Psychiatry, Charité - Universitätsmedizin Berlin, Campus Benjamin Franklin, Hindenburgdamm 30, 12203 Berlin, Germany; 4 Department of Child and Adolescent Psychiatry, University Medical Center Göttingen, Von-Siebold-Str. 5, 37075 Göttingen, Germany; 5 Department of Child and Adolescent Psychiatry, Medical Faculty of the Technical University Dresden, Fetscherstr. 74, 01307 Dresden, Germany; 6 Department of Child and Adolescent Psychiatry, LVR-Klinikum Düsseldorf/ Heinrich-Heine University Düsseldorf, Bergische Landstr. 2, 40629 Düsseldorf, Germany

**Keywords:** Autism spectrum disorder, Autism, Complementary and alternative medicine, CAM, Prevalence, Adults, Germany

## Abstract

**Background:**

Complementary and Alternative Medicine (CAM) is widely used both in the general population and for the treatment of somatic and psychiatric disorders. Studies on CAM use among patients with autism spectrum disorder (ASD) have so far only focused on children and adolescents. The aim of this study was to investigate patterns of CAM use among adults with ASD.

**Methods:**

A questionnaire survey concerning current and lifetime use of CAM was distributed to adults with ASD between November 2015 and June 2016. Participants diagnosed by experienced clinicians using the current diagnostic gold standard were recruited from four ASD outpatient clinics in Germany. Questionnaire data was then linked to supplementary clinical data.

**Results:**

The final sample consisted of 192 adults (response: 26.8%) with a mean age of 31.5 years (80% male; diagnoses: Asperger’s syndrome (58%), childhood autism (27%), atypical autism (12%)). 45% of the respondents stated that they were currently using or had used at least one CAM modality in their life. Among the participants with lifetime CAM use, almost half had used two or more different types of CAM. Alternative medical systems (e.g. homeopathy, acupuncture) were most frequently used, followed by mind-body interventions (e.g. yoga, biofeedback, animal assisted therapy). Overall, 20% of respondents stated that they would like to try at least one listed CAM modality in the future.

**Conclusions:**

This is the first study on CAM use in adults with ASD, demonstrating considerable CAM use in this population. Given the popularity of CAM, patients should be informed about the effectiveness and potentially dangerous side effects of CAM treatments, as evidence for the majority of CAM methods in ASD is still limited.

## Introduction

The term “Complementary and Alternative Medicine (CAM)” denotes a heterogeneous group of diverse medical and health care practices that are not considered to be part of conventional health care systems [1]. Complementary medicine is typically defined as nontraditional practices that are used together with conventional medicine, whereas alternative medicine is used in place of conventional medicine [2].

The utilisation of CAM is widespread, both in healthy individuals and those with somatic or psychiatric disorders. In a systematic review of 49 studies from 15 countries, the prevalence of CAM use in the general population ranged from 10 to 76% [3], with the large variance being due to geographical, economic, social, cultural and methodological factors. However, several studies have demonstrated that typical CAM users are likely to be female, better educated and to have a higher income [4–9]. CAM therapies are also used for the treatment of many physical as well as mental disorders, and especially those of a more chronic nature, e.g. cancer, asthma, depression or autism spectrum disorders (ASD) [10]. Compared to the general population, the utilization of CAM is higher amongst individuals with these disorders [4, 5, 7, 11–14].

ASD is a neurodevelopmental disorder which is characterized by pervasive difficulties in interaction and communication that are accompanied by unusually restricted, repetitive behavior and interests since early childhood [15, 16]. It is a high-cost and lifelong condition that affects up to 1% of adults [17]. For children, the prevalence was 16.8 per 1000 children in 2014 [18], with prevalences steadily increasing in recent decades [19–21]. To date, no causal treatment for ASD is known, but there exist various medical, behavioral and educational interventions that aim to mitigate the core deficits related to the disorder, and to improve psychiatric comorbidity [16]. Comorbid symptoms of ASD such as hyperactivity, anxiety, aggression, or insomnia, are frequently treated with both conventional therapies, including psychopharmacological agents, and CAM therapies [10, 22].

While there is limited evidence for the effectiveness of some types of CAM for the treatment of ASD core symptoms and associated comorbidities [23, 24], most CAM treatments have not yet been adequately studied for this indication [25].

Although many CAM modalities are noninvasive and free from side effects (e.g. yoga or music therapy), there are some types of CAM, such as chelating agents or megavitamin therapy, for which safety concerns exist due to potentially dangerous side effects. Furthermore, knowledge on pharmacological interaction effects between CAM therapies and psychotropic drugs is scarce [26–29], and some treatments (e.g. special diets) might be associated with potentially harmful, long-term side effects, such as nutritional deficits [25].

Despite the limited evidence for CAM use in ASD, a recent systematic review yielded relatively high prevalences of CAM use in children and adolescents with ASD, ranging from 28 to 95% (median: 54%), with special diets or dietary supplements (including vitamins) being the most frequent CAM treatments [10]. However, none of the surveys covered by the afore-mentioned review was conducted in adults with ASD. Therefore, the aim of the present study was to investigate prevalence and type of CAM use among adults with a diagnosis of ASD.

## Methods

This study was conducted as a part of a large clinical and research network, the ASD-Net, which is funded by the German federal ministry of education and research (BMBF), and which focuses on the key challenges in ASD diagnostics, therapy and health service research [21].

### Recruitment and participants

Data for this study was collected in four academic ASD outpatient clinics in Germany (Berlin, Dresden, Mannheim, and Marburg). Between November 2015 and June 2016, these study centers recruited adults with an ASD diagnosis, who received services from or had been diagnosed at one of these outpatient clinics. Patients were included if they were 18 years or older, and had a confirmed diagnosis of ASD according to ICD-10 (F84.0, F84.1, F84.5, F84.8, F84.9). Regarding the classification of ASD diagnoses in this study, it should be noted that, unlike the DSM-5, the ICD-10 has not incorporated the concept of autism as a “spectrum disorder”, and therefore offers different diagnostic categories for patients with autism (e.g. “atypical autism” (F84.1), which is equivalent to PDD-NOS in DSM-IV (299.80)). All participants were diagnosed by experienced clinicians using the current diagnostic gold standard in ASD, the Autism Diagnostic Observation Schedule (ADOS) and – if parents were available – the Autism Diagnostic Interview-Revised (ADI-R) [21, 30, 31].

### Questionnaire and data collection

Based on the Client Service Receipt Inventory (CSRI) [32], a self-administered questionnaire on sociodemographic data (including educational level), and health and social service use, including CAM, was developed and has been mailed to the participants. In exceptional cases, the questionnaire was handed over personally. Each questionnaire was accompanied by a cover letter, a participant information sheet and a written informed consent form, in which participants could consent to data linkage between their questionnaire data and their clinical data (e.g. age, sex, ICD 10-diagnosis, IQ).

The questionnaire item on CAM use was configured as follows: Following the classification of the National Center for Complementary and Integrative Health (NCCIH), we listed five CAM categories: [1] Alternative medical systems or complete systems of therapy and practice (e.g. acupuncture), [2] mind-body interventions (e.g. yoga), [3] biologically based therapies (e.g. diets), [4] manipulative and body based methods (e.g. craniosacral therapy), and [5] other CAM practices (e.g. quigong) [33]. For each of the five categories (a non-exhaustive list of examples was provided), participants could mark one of the following response options: “Yes, I use this CAM category currently”, “Yes, I used in the past”, “I would like to try it”, or “No”.

Participants’ level of education was defined in accordance with the International Standard Classification of Education (ISCED) [34, 35], and classified into three groups: low (ISCED level 0–2B), medium (level 2A) and high education (level 3A). Referring to the German school system, low educational level complies with 9 years of schooling or leaving school without having acquired any school-leaving qualification. Medium educational level is equivalent to 10 years of schooling, and high educational level complies with 12 or 13 years of schooling and a school-leaving qualification, which opens access to higher education institutions [36, 37].

The IQ was assessed using the German versions of the following instruments: Wechsler Intelligence Scale for Children (WISC-R [38] WISC-III [39], WISC-IV [40]), Wechsler Adult Intelligence Scale (WAIS-R [41], WAIS-III [42]) Wechsler Preschool and Primary Scale of Intelligence (WPPSI-III [43]), Kaufman Assessment Battery for Children [44], Wortschatztest [45], Raven’s Standard Progressive Matrices [46], and Raven’s Coloured Progressive Matrices [47]. According to ICD-10, the level of intellectual functioning was classified into two groups: learning disability or intellectual disability (IQ < 85) vs no learning disability or intellectual disability (IQ ≥ 85).

Data from the questionnaires were entered by one person in an electronic Case Report Form (eCRF) created in OpenClinica® (OpenClinica Enterprise Version: 3.3), and were checked by a second person.

Baseline data were analysed using descriptive statistics. The prevalence of CAM use in the study population was stratified by age (18–24, 25–34, ≥35 years), sex (male, female), ASD subgroup (Asperger syndrome, other ASD diagnoses), intellectual functioning (IQ < 85, IQ ≥ 85) and educational level (low, medium, high).

To calculate 95% confidence intervals (95% CIs), the Clopper-Pearson Exact method was applied. Additionally, the association between CAM use and the above mentioned predictors was evaluated in a logistic regression model; with odds ratios (OR) and 95% CIs being inferred. All statistical analyses were performed with SAS, version 9.4 (SAS Institute, Cary, USA).

The study protocol was reviewed and approved by the Commission for Research Impact Assessment and Ethics, Carl von Ossietzky University Oldenburg, and by the respective ethic committees of the participating study sites.

## Results

### Baseline characteristics

Survey documents were sent to 782 adults with ASD. In 52 cases, mailings could not be delivered due to a wrong address. Two hundred and six persons returned the questionnaire including a signed written consent form (response: 26.8%), but in 10 cases clinical data for linkage was not available. Of the remaining 196 participants, 192 answered at least one CAM-related question and were thus included as study population. 22.2% of the adult non-responders and 31.8% of the responders were aged ≥35 years. The baseline characteristics of the study population are presented in Table 1. Most of the participants were male (79.7%), and the mean age was 31.5 years (range: 18–67 years). The most frequent diagnosis was Asperger syndrome (57.8%), followed by childhood autism (27.1%), and atypical autism (12.0%). More than two thirds of the study population were of average or above average intelligence (69.7%), and nearly half had a high level of education (44.3%).

**Table 1:** Baseline characteristics

Characteristics (number of respondents^a^)	n	%
Sex (*n* = 192)		
Male	153	79.7
Female	39	20.3
Mean age (in years) (±SD; range)	31.5 (10.9; 18–67)	
Age groups (in years) (n = 192)		
18–24	59	30.7
25–34	72	37.5
≥ 35	61	31.8
Diagnoses (n = 192)		
Childhood autism (F84.0)	52	27.1
Atypical autism (F84.1)	23	12.0
Asperger syndrome (F84.5)	111	57.8
Other specified pervasive developmental disorders (F84.8)	1	0.5
Pervasive developmental disorder, not otherwise specified (F84.9)	5	2.6
Intellectual functioning (*n* = 178)		
IQ < 85	54	30.3
IQ ≥ 85	124	69.7
Level of education (n = 192)		
Low	56	29.2
Medium	51	26.6
High	85	44.3

^a^due to missing values, figures may differ

### Overall CAM use

Overall, 44.8% of the respondents stated that they were currently using, or had used at least one CAM modality in their life (Table 2). Almost 30% of the sample reported current CAM use, and 24.5% indicated that they had used some type of CAM in the past.

**Table 2:** Stratified prevalence of lifetime, current, and past CAM use, and of willingness to try CAM

		Prevalence in % (95% CI)		
	lifetime use^a^	current use	past use	willingness to try^b^
Sex				
Male	43.8 (35.8–52.0)	27.5 (20.6–35.2)	24.2 (17.6–31.8)	20.3 (14.2–27.5)
Female	48.7 (32.4–65.2)	33.3 (19.1–50.2)	25.6 (13.0–42.1)	17.9 (7.5–33.5)
Age groups (in years)				
18–24	44.1 (31.2–57.6)	20.3 (11.0–32.8)	32.2 (20.6–45.6)	10.2 (3.8–20.8)
25–34	40.3 (28.9–52.5)	30.6 (20.2–42.5)	18.1 (10.0–28.9)	20.8 (12.2–32.0)
≥ 35	50.8 (37.7–63.9)	34.4 (22.7–47.7)	24.6 (14.5–37.3)	27.9 (17.1–40.8)
Diagnoses				
Other ASD diagnosis	42.0 (31.1–53.5)	28.4 (18.9–39.5)	22.2 (13.7–32.8)	21.0 (12.7–31.5)
Asperger syndrome	46.8 (37.3–56.6)	28.8 (20.6–38.2)	26.1 (18.2–35.3)	18.9 (12.1–27.5)
Intellectual functioning				
IQ < 85	42.6 (29.2–56.8)	31.5 (19.5–45.6)	25.9 (15.0–39.7)	16.7 (7.9–29.3)
IQ ≥ 85	42.7 (33.9–51.9)	27.4 (19.8–36.2)	21.0 (14.2–29.2)	21.0 (14.2–29.2)
Level of education				
Low	44.6 (31.3–58.5)	32.1 (20.3–46.0)	25.0 (14.4–38.4)	17.9 (8.9–30.4)
Medium	41.2 (27.6–55.8)	23.5 (12.8–37.5)	27.5 (15.9–41.7)	9.8 (3.3–21.4)
High	47.1 (36.1–58.2)	29.4 (20.0–40.3)	22.4 (14.0–32.7)	27.1 (18.0–37.8)
Overall	44.8 (37.6–52.1)	28.6 (22.4–35.6)	24.5 (18.6–31.2)	19.8 (14.4–26.1)

^a^contains all respondents who stated current use, past use or both

^b^contains also respondents who stated that they already use or have used some other CAM modality

The three types of CAM use prevalence (lifetime, current, past), stratified by sex, age, clinical diagnosis, intellectual functioning, and level of education are presented in Table 2. Regarding lifetime prevalence, participants aged 35 years or older (50.8%), were more likely to have used CAM, compared to those younger than 35 years (44.1% vs. 40.3%, respectively). Females (48.7%), and patients with a diagnosis of Asperger syndrome (46.9%) reported slightly more frequent lifetime use of CAM than males (43.8%) respectively than patients with other ASD diagnoses (42.0%). Stratified by educational level, lifetime use was highest in patients with a high school-leaving qualification (47.1%). Stratification of lifetime CAM use by intellectual functioning did not reveal any differences.

In a multivariate logistic regression using sex, age, type of diagnosis, intellectual functioning, and educational level as predictors of lifetime CAM use, none of the predictors were significant.

### CAM modalities

Among the participants with lifetime CAM-use, almost half (46.5%) had used treatments from two or more different categories of CAM. As shown in Table 3, the most commonly used categories were “alternative medical systems or complete systems of therapy and practice” (26.9%), e.g. homeopathy, acupuncture or Traditional Chinese Medicine, and “mind-body interventions” (23.6%), e.g. yoga, music therapy, biofeedback, or animal assisted therapy.

**Table 3:** Prevalence of lifetime, current, past use and willingness to try different CAM categories

	Prevalence in % (95% CI)			
	lifetime use	current use	past use	willingness to try
CAM group 1: Alternative Medical Systems or complete systems of therapy and practice *(*e.g. *Anthroposophic medicine, homeopathy, naturopathic medicine, Traditional Chinese Medicine, acupuncture)*	26.9 (20.7–33.9)	10.8 (6.7–16.1)	16.1 (11.2–22.2)	4.8 (2.2–9.0)
CAM group 2: Mind-Body Interventions *(*e.g. *yoga, biofeedback, music therapy, dance therapy, meditation, animal-assisted therapy,* prayer *and spiritual healing*)	23.6 (17.7–30.5)	13.2 (8.6–19.0)	10.4 (6.4–15.8)	9.9 (6.0–15.2)
CAM group 3: Biologically Based Therapies *(*e.g. *special diets (*e.g. *gluten free, casein free), dietary supplements, melatonin, naltrexone, vitamin C, vitamin B6/magnesium, amino acids, omega 3 fatty acids, trace elements, secretin, chelation therapy, hyperbaric oxygen therapy)*	17.6 (12.3–24.1)	14.2 (9.4–20.3)	3.4 (1.3–7.3)	5.1 (2.4–9.5)
CAM group 4: Manipulative and Body Based Methods *(*e.g. *facilitated communication, PECS, chiropractic, osteopathy, craniosacral therapy, sensory integration therapy or auditory integration training, Bobath)*	15.3 (10.4–21.5)	6.3 (3.2–10.9)	9.1 (5.3–14.3)	10.2 (6.2–15.7)
CAM group 5: Other CAM Practices *(*e.g. *energy medicine, Feldenkrais, Pilates, Qigong, Reiki*)	3.5 (1.3–7.5)	1.8 (0.4–5.0)	1.8 (0.4–5.0)	11.1 (6.8–16.8)

*CAM* complementary and alternative medicine, *CI* confidence interval, *PECS* picture exchange communication system

Regarding current CAM use, the modality most frequently used was “Biologically Based Therapies” (14.2%), while “Alternative medical systems or complete systems of therapy and practice” (16.1%) were the most common modality in the past use category.

Regarding lifetime use, no notable differences between the five CAM categories according to sex, age, diagnosis, intellectual functioning, or educational level were found (Table 4).

**Table 4:** Stratified prevalence of lifetime use of different CAM categories

	Lifetime use in % (95% CI)				
	CAM category 1	CAM category 2	CAM category 3	CAM category 4	CAM category 5
Sex					
Male	26.5 (19.6–34.4)	25.9 (19.0–33.7)	15.7 (10.1–22.8)	15.0 (9.5–22.0)	2.9 (0.8–7.4)
Female	28.2 (15.0–44.9)	14.3 (4.8–30.3)	25.0 (12.1–42.2)	16.7 (6.4–32.8)	5.7 (0.7–19.2)
Age groups (in years)					
18–24	31.6 (19.9–45.2)	23.2 (13.0–36.4)	10.7 (4.0–21.9)	14.3 (6.4–26.2)	0 (0.0–0.0)
25–34	26.5 (16.5–38.6)	23.5 (14.1–35.4)	17.2 (8.9–28.7)	13.8 (6.5–24.7)	1.6 (0.0–8.8)
≥ 35	23.0 (13.2–35.5)	24.1 (13.9–37.2)	25.0 (14.4–38.4)	18.2 (9.1–30.9)	8.9 (3.0–19.6)
Diagnoses					
Other ASD diagnosis	29.5 (19.7–40.9)	27.0 (17.4–38.6)	18.3 (10.1–29.3)	19.7 (11.2–30.9)	3.0 (0.4–10.4)
Asperger syndrome	25.0 (17.2–34.3)	21.3 (14.0–30.2)	17.1 (10.5–25.7)	12.4 (6.8–20.2)	3.8 (1.1–9.6)
Intellectual functioning					
IQ < 85	34.0 (21.5–48.3)	25.0 (13.6–39.6)	18.8 (8.9–32.6)	20.8 (10.5–35.0)	2.2 (0.1–11.8)
IQ ≥ 85	23.8 (16.5–32.3)	19.8 (13.1–28.1)	16.9 (10.7–25.0)	12.7 (7.3–20.1)	3.4 (0.9–8.5)
Level of education					
Low	32.1 (19.9–46.3)	31.4 (19.1–45.9)	18.4 (8.8–32.0)	22.4 (11.8–36.6)	4.4 (0.5–15.2)
Medium	26.5 (14.9–41.1)	24.5 (13.3–38.9)	15.2 (6.3–28.9)	17.0 (7.6–30.8)	4.3 (0.5–14.8)
High	23.8 (15.1–34.4)	18.3 (10.6–28.4)	18.5 (10.8–28.7)	10.0 (4.4–18.8)	2.5 (0.3–8.7)
Overall	26.9 (20.7–33.9)	23.6 (17.7–30.5)	17.6 (12.3–24.1)	15.3 (10.4–21.5)	3.5 (1.3–7.5)

### Willingness to try CAM

Of the 192 participants, 19.8% stated that they would like to try at least one listed CAM modality in the future, including respondents who stated that they already use or had used another CAM method listed in the questionnaire. 6.3% of the respondents had not yet used any type of CAM but would like to try it. Willingness to try CAM increased with higher age (10.2% vs. 20.8% vs. 27.9%, respectively), and respondents with a high level of education (27.1%) were more frequently interested in trying CAM than those with a lower educational level (medium: 9.8%, low: 17.9%).

## Discussion

In this study, 44.8% of the responding adults with ASD stated that they are currently using or had used at least one CAM modality in their life.

As this study is the first of its kind, its results can only be compared to studies with non-autistic adults. As mentioned in the introduction, the prevalence of CAM use in the general population is estimated to be between 10 and 76% [3]. For Germany, a systematic review found the prevalence of CAM use ranging from 40 to 62% in the general population [48].

Regarding CAM use in psychiatric disorders in general, de Jonge et al. [49] studied the prevalence of “CAM contact” in adults with a range of mental disorders in 25 high-income countries (*N* = 138,801), drawing on representative data from the World Mental Health Surveys (studied period: 2001–2012). In their study, they found an overall CAM use prevalence of 4.6%. When including only those individuals with severe behavioral disorders receiving specialist psychiatric care, CAM use increased to 22.5%. However, de Jonge et al. used a different, possibly narrower, definition of CAM which could account for the lower percentage of usage found in their study. Still, this figure is only half of the prevalence measured in this study. Nevertheless, other population-based studies from western countries on CAM use for the treatment of psychiatric disorders found higher utilisation: A large review that included 45 publications on CAM use in patients with depression found a prevalence 10–30% in depressive disorder, and of 20–50% in bipolar disorder [50]. In a Finnish study in adults aged 30 years and older, participants with anxiety disorder reported a CAM utilisation prevalence (last 12 months) of 45% [51], while a representative study from the UK found a 12-months-CAM utilisation prevalence of 35% in respondents with anxiety or depression. In comparison to the afore-mentioned studies, our findings lie in the upper half of the reported range. The comparably high prevalence of CAM use in ASD may reflect the presently “incurable” nature of ASD core symptoms, in contrast to other psychiatric disorders where the core symptoms might be addressed successfully through e.g. pharmacotherapy or psychotherapy.

Concerning CAM use in children and adolescents with ASD, there exists a considerable body of literature. A systematic review of 20 studies with a total of 9540 participants found CAM use ranging from 28 to 95% in children and adolescents [10], with special diets and dietary supplements being most frequently utilized. In a more recent German survey of caregivers of children and adolescents with ASD, 46.3% acknowledged current or lifetime CAM use [52]. The similarity between CAM use in children and adolescents, and in adults is noteworthy, as one might expect higher CAM use in adults, who have a higher degree of freedom to decide which therapeutic options to use.

It is also worth comparing the CAM use prevalence found in this study with the prevalence of psychopharmacotherapy utilisation in adults with ASD. The most comprehensive review so far, by Jobski et al. [22], analysed 47 studies from a time period of more than 30 years, including more than 300,000 patients with ASD. In this review, the median for psychopharmacotherapy utilisation in adults with ASD was 61.5%, thus lying clearly above the figure for CAM use in our study.

Regarding potential predictors of CAM use, in our study none of these showed a significant association with either lifetime or current CAM utilisation. The reason for this lack of predictors, which differs from other studies, which found e.g. female gender and higher education to be predictors for CAM use, is not clear. Probably both the relatively small sample size and the selection bias of the sample, namely a significant portion of patients with a diagnosis of Asperger syndrome, have contributed to these inconclusive findings.

As to the CAM modalities being used most frequently, in this study alternative medical systems (e.g. homeopathy, acupuncture), and mind-body interventions (e.g. yoga, music therapy) were the leading modalities. This stands in contrast to CAM use in children and adolescents with ASD, where – as mentioned above – special diets and dietary supplements are the most frequently utilized CAM modality. This difference may be explained by the fact that the postulated mode of action for diets in ASD often addresses improvements within the developing brain, which is more attractive for parents of children with ASD than for affected adult individuals. Moreover, intervention like yoga are often better accessible for adults than for children. Nevertheless, it has to be kept in mind that the term “CAM” denotes a very heterogeneous group of modalities, with different studies using differing definitions of CAM subgroups, thus making a clear delimitation of CAM subgroups difficult.

Finally, this study found 19.8% of respondents being willing to try CAM in the future. This figure is difficult to interpret, as it is unclear whether this reflects a positive attitude towards CAM without prior CAM experiences, or discontent with prior CAM use in 80% of respondents.

As mentioned above, the relatively high CAM utilisation prevalence found in this study possibility reflects at least partly the burden caused by the reduced quality of life of individuals with ASD, and the lack of a causal treatment for this condition. In this context, affected individuals might perceive CAM treatments as a last resort. To help them avoid choosing potentially harmful CAM treatments, guidelines for the management of ASD [53] should offer both professionals and patients clear information on effective, non-effective, and even harmful treatments.

### Strengths and limitations

To our knowledge, this study is the first survey ever to evaluate CAM use in a population of adults with ASD, which constitutes a major strength. Moreover, the underlying sample includes a reasonable mixture of ASD subtypes, with high diagnostic quality.

Nevertheless, this study also has several limitations: First, CAM use was evaluated through a self-report questionnaire. Still, using self-report instruments on CAM use may induce more honest answers than face-to-face interviews [54]. Second, because of the (for the sake of brevity) non-exhaustive list of CAM modalities presented in the questionnaire, CAM use may have been underestimated. Third, respondents’ reports of lifetime and past CAM use may have been impacted by recall bias. Fourth, the questionnaire employed did not ask about patients’ satisfaction or symptom improvement with CAM treatments [49]. Fifth, in view of age differences between respondents and non-respondents, selection bias cannot be excluded. However, regarding CAM use we did not find any differences by age. Moreover, we did not evaluate respondents’ medication or comorbidities. Finally, patients for this study were recruited at four highly specialised outpatient clinics, so the sample composition may not be representative for the population of adult ASD patients in Germany in general.

## Conclusion

This is the first study on CAM use in adults with autism spectrum disorder, demonstrating a considerable prevalence of CAM use in this population. Considering its popularity, health professionals should be aware of the significant prevalence of CAM use in adults with ASD, especially as CAM use is often not disclosed to physicians. Also, patients should be encouraged to critically evaluate information about the effectiveness and potentially dangerous side effects of CAM treatments, as evidence for the majority of these treatments in ASD is still limited.

## Abbreviations

ADI-R: Autism diagnostic interview – revised
ADOS: Autism diagnostic observation schedule
ASD: Autism spectrum disorder
CAM: Complementary and alternative medicine
CI: Confidence interval
CSRI: Client service receipt inventory
eCRF: electronic case report form
ICD-10: International statistical classification of diseases and related health problems - 10th revision
ID: Intellectual disability
ISCED: International standard classification of education
LD: Learning disability
NCCAM: National center for complementary and integrative health
OR: Odds ratio
PDD-NOS: Pervasive developmental disorder - not otherwise specified
SAS: Statistical analysis software
SD: Standard deviation
UK: United Kingdom
USA: United States of America
